# A pre-anesthetic bolus of ketamine versus dexmedetomidine for prevention of postoperative delirium in elderly patients undergoing emergency surgery: a randomized, double-blinded, placebo-controlled study

**DOI:** 10.1186/s12871-023-02367-8

**Published:** 2023-12-11

**Authors:** Huda F. Ghazaly, Tarek S. Hemaida, Zaher Z. Zaher, Omar M. Elkhodary, Soudy S. Hammad

**Affiliations:** Anesthesia and Surgical Intensive Care Department, Faculty of Medicine, Aswan University, Aswan, Egypt

**Keywords:** Dexmedetomidine, Elderly patients, Emergency surgery, Ketamine, Postoperative delirium

## Abstract

**Background:**

We aimed to evaluate whether a single dose of ketamine or dexmedetomidine before induction of general anesthesia could reduce the incidence of postoperative delirium (primary outcome) or cognitive dysfunction (secondary outcome) in elderly patients undergoing emergency surgery.

**Patients and methods:**

This randomized, double-blinded, placebo-controlled trial included 60 elderly patients who were scheduled for emergency surgery. The patients were randomly assigned into one of three groups (n = 20): group I received 0.9% normal saline, group II received 1 µg/kg dexmedetomidine, and group III received 1 mg/kg ketamine right before anesthesia induction. Patients were observed for three days after surgery and tested for postoperative delirium and cognitive dysfunction using the delirium observation screening scale and the mini-mental state examination score, respectively.

**Results:**

The dexmedetomidine group had the lowest incidence of delirium (p = 0.001) and cognitive dysfunction (p = 0.006) compared to the ketamine and placebo groups. The multivariate logistic regression model revealed that dexmedetomidine reduced the incidence of postoperative delirium by 32% compared to placebo (reference) (OR = 0.684, 95% CI: 0.240–0.971, p = 0.025), whereas ketamine increased the risk by threefold (OR = 3.012, 95% CI: 1.185–9.681, p = 0.013). Furthermore, dexmedetomidine reduced the incidence of postoperative cognitive dysfunction by 62% (OR = 0.375, 95% CI: 0.091–0.543, p = 0.012), whereas ketamine increased the risk by 4.5 times (OR = 4.501, 95% CI: 1.161–8.817, p = 0.006).

**Conclusion:**

A single pre-anesthetic bolus of dexmedetomidine is a practical choice for preventing postoperative delirium in elderly patients undergoing emergency surgery.

**Trial registration:**

This study was approved by the Ethics Committee of Aswan University Hospital (approval number: aswu/548/7/2021; registration date: 06/07/2021) and registered on ClinicalTrials.gov (NCT05341154) (22/04/2022).

## Introduction

Delirium is an acute, fluctuating mental state characterized by impaired awareness and attention disturbance that develops over a short period (usually hours to days). Postoperative delirium (POD) is a serious postoperative complication that can last up to 5 days after surgery and has negative consequences such as increased mortality and financial cost. POD is frequently associated with deteriorating cognition, known as postoperative cognitive dysfunction (POCD) [1]. Even though the pathophysiology of delirium remains poorly understood, many studies have identified advanced age and emergency surgery as risk factors for POD. Approximately 80% of elderly people undergoing surgery develop POD, which is 1.5 to three times more likely after emergency surgery than non-emergency surgery [2–4]. Thus, preventive interventions are especially important since they form the foundation for POD management [5]. A variety of pharmacological therapies are utilized to prevent or minimize POD [6]. Dexmedetomidine is an alpha-2 adrenoceptor agonist with analgesic and neuroprotective effects. These neuroprotective characteristics may explain the growing evidence of the preventative effect of dexmedetomidine on POD [7]. Similarly, ketamine is an N-methyl-D-aspartate antagonist that has been demonstrated to have neuroprotective benefits in a variety of experimental tests, mostly through its ability to inhibit apoptosis and inflammation [8]. As a result, clinical trials are increasingly being utilized to assess the efficacy of ketamine on POD and neurocognition. Nevertheless, POD outcomes are still uncertain due to discrepancies in the interventions used to reduce them. There is a need for a consensus on the most effective way to prevent POD, especially among elderly people, whose incidence and prevalence rates are higher, and thereby alleviate the financial burden associated with POD.

This randomized, double-blinded, placebo-controlled trial aimed to evaluate whether a single dose of ketamine or dexmedetomidine before induction of general anesthesia could reduce postoperative delirium incidence (primary outcome), postoperative cognitive dysfunction, postoperative pain, and opioid consumption (secondary outcome) in elderly patients undergoing emergency surgery.

## Patients and methods

### Ethics and registration

This study was approved by the Ethics Committee of Aswan University Hospital (approval number: aswu/548/7/2021; registration date: 06/07/2021) and registered on ClinicalTrials.gov (NCT05341154) (22/04/2022). It was held between May 2022 and May 2023 and followed the Consolidated Standards of Reporting Trials guidelines. The study’s objectives and risks were explained to participants, and they signed written informed consent. All methods were carried out according to the Helsinki Declaration of 1964 and its amendments.

### Patient inclusion and exclusion criteria

The study included all patients aged 60 or older with an ASA I or II who were scheduled for emergency gastrointestinal, orthopedic, vascular, obstetric, urologic, or plastic surgery. Patients with preoperative delirium determined by the Delirium Observation Screening Scale (DOSS) [9] or cognitive impairment as measured by the Mini-Mental State Examination (MMSE) scores [10], a history of dexmedetomidine or ketamine intolerance, a lack of collaboration or communication, Parkinson’s disease, epilepsy, or a high probability of postoperative care in the intensive care unit (ICU) were excluded from the study.

### Randomization

Computer-generated randomization tables were used to establish randomization. The group assignment was concealed in serially numbered, sealed, opaque envelopes. Patients were assigned at random to one of three equal groups (n = 20): group I (placebo group) received 0.9% normal saline, group II (dexmedetomidine group) received 1 µg/kg dexmedetomidine, and group III (ketamine group) received 1 mg/kg ketamine. An investigator who was not involved in patient anesthesia or outcome evaluation prepared the study drug in a standardized syringe with the same volume (50 ml) for all groups. The patients, the researcher who performed anesthesia, and the data collectors were blinded to the group assignment.

### Anesthesia

Before proceeding to the operating room, the authors assessed the patient’s eligibility for the study through history-taking, physical examination, and evaluation of basal laboratory studies. Standard monitoring was connected upon arrival in the operating room, including ECG, non-invasive arterial blood pressure, and pulse oximetry. All patients received the study drug over 10 min right before anesthesia induction, followed by a consistent general anesthetic regimen. Fentanyl (1 µg/kg body weight) was used for analgesia, propofol (1–2 mg/kg body weight) was used to induce anesthesia, rocuronium (1 mg/kg body weight) was used to allow insertion of the endotracheal tube, and isoflurane in 50% O_2_ / air was used to maintain anesthesia. The mechanical ventilation was set to keep the end-tidal carbon dioxide partial pressure (EtCO_2_) level between 35 and 40 mmHg. Isoflurane was stopped at the end of the procedure, and the residual neuromuscular block was reversed with neostigmine and atropine.

### Outcome measures

The primary endpoint was the incidence of delirium within three days after surgery. Patients were examined for delirium twice a day (after 6.00 a.m. and again after 6.00 p.m.). The secondary outcomes included postoperative cognitive dysfunction, duration of surgery (measured from incision to wound closure), duration of anesthesia (measured from anesthesia induction to anesthetic drug discontinuation), time to extubation (measured from anesthetic drug discontinuation to extubation), and the total doses of fentanyl used during the operation. Postoperative pain was assessed by the Visual Analog Scale (VAS) (0 = no pain, 10 = most severe pain) at the 30th minute (t1), 1st hour (t2), 4th hour (t3), 12th hour (t4), and 24th hour (t5) after surgery. The time to first postoperative request for analgesia (nalbuphine 0.05 mg/kg) when VAS was ≥ 3 was recorded, and the total nalbuphine consumption in the first 24 h was calculated.

### Sample size and statistical analysis

The sample size was calculated using G*Power 3 software, with a power of 80% and a type I error of 5% (α = 0.05 and β = 80%) on the one-tailed test, to detect an effect size of 0.4 to 0.5 in the incidence of postoperative delirium, which is the primary outcome measure of this study. The minimum required sample size was 60 patients admitted and scheduled for emergency surgery under general anesthesia (divided into three equal groups of 20 patients each).

The data were verified, coded by the researchers, and analyzed using SPSS version 24. Descriptive statistics: means, standard deviations (SD), and percentages were calculated. The chi-square test was used to compare the difference in the distribution of postoperative delirium incidence among different groups. The Shapiro-Wilk test was used to test data normality. For continuous variables with more than two categories, the one-way analysis of variance (ANOVA) test was calculated to test the mean differences of the data that follow a normal distribution, and an independent post-hoc test was calculated using Bonferroni corrections. The multivariate logistic regression analysis was used to detect the independent association between treatment drugs and the incidence of postoperative delirium and cognitive dysfunction. A p-value equal to or less than 0.05 was considered significant.

## Results

**Fig. 1:**
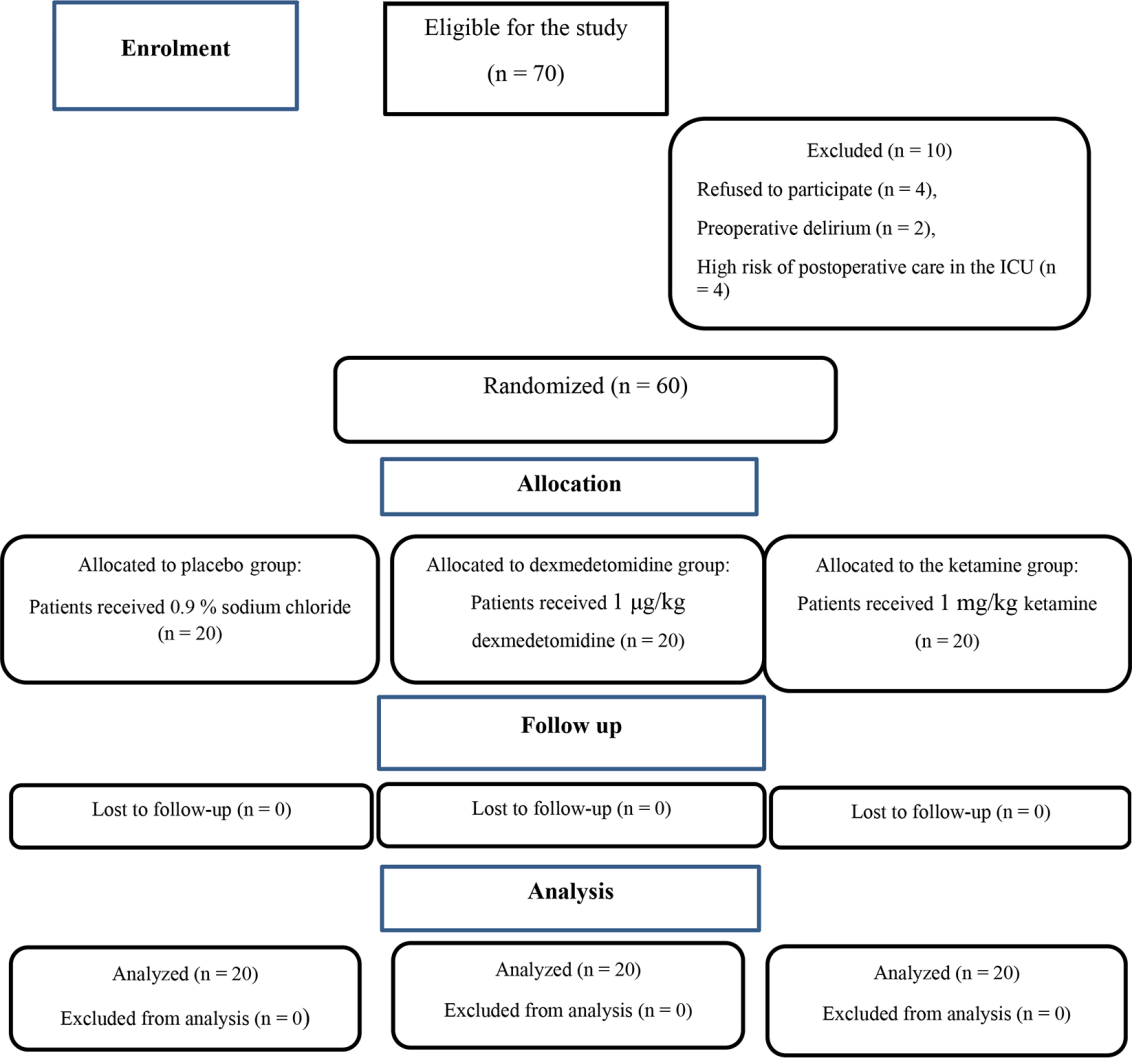
CONSORT flow diagram of study participants

Seventy patients were eligible for this study. The authors excluded ten patients because four refused to participate and six did not meet the inclusion criteria (two due to preoperative delirium, and four were at high risk of postoperative care in the ICU). Finally, 60 patients were enrolled in this study based on intention to treat and randomly assigned to one of three equal groups (Fig. 1). The baseline patient characteristics and laboratory studies were comparable across the study groups. In terms of surgical data, the type of surgery and total fentanyl consumption during the operation were not significantly different between groups, but the length of surgery, duration of anesthesia, and time to extubation were significantly shorter in the ketamine group compared to the dexmedetomidine and placebo groups (Table 1).

**Table 1:** Baseline patient characteristics and surgical data

	Placebo group (n = 20)	Dexmedetomidine group (n = 20)	Ketamine group (n = 20)	P-value	
Age/year	69.41 ± 4.7	70.01 ± 3.8	71.15 ± 3.9	= 0.371	
P-value	I vs. II = 0.247	II vs. III = 0.910	I vs. III = 0.205		
Sex				= 0.262	
Female	8 (40%)	5 (25%)	10 50%)		
Male	12 (60%)	15 (75%)	10 (50%)		
BMI (kg/m^2^)	26.91 ± 2.8	25.96 ± 2.2	27.09 ± 2.1	= 0.278	
P-value	I vs. II = 0.211	II vs. III = 0.137	I vs. III = 0.810		
ASA					
I	17 (85%)	16 (80%)	14 (70%)	= 0.503	
II	3 (15%)	4 (20%)	6 (30%)		
Type of surgery				= 0.884	
Orthopaedic	8 (40%)	4 (20%)	5 (25%)		
Plastic	1 (5%)	1 (5%)	1 (5%)		
Urologic	2 (10%)	3 (15%)	2 (10%)		
Gastrointestinal	9 (45%)	12 (60%)	12 (60%)		
Length of surgery (min.)	95.40 ± 20.1	95.01 ± 18.4	69.01 ± 16.3	< 0.001	
P-value	I vs. II = 0.946	II vs. III < 0.001	I vs. III < 0.001		
Anesthesia duration (min.)	121.75 ± 19.5	121.70 ± 20.4	90.75 ± 18.2	< 0.001	
P-value	I vs. II = 0.994	II vs. III < 0.001	I vs. III < 0.001		
Extubation time (min.)	14.25 ± 2.7	12.80 ± 3.4	10.10 ± 2.8	< 0.001	
P-value	I vs. II = 0.130	II vs. III = 0.006	I vs. III < 0.001		
Total fentanyl dose during operation (µg)					
	85.01 ± 17.8	81.25 ± 14.5	86.25 ± 17.2	= 0.813	
P-value	I vs. II = 0.644	II vs. III = 0.538	I vs. III = 0.878		

ASA, American Society of Anesthesiologists, BMI; Body Mass Index

### Primary and secondary outcomes

The incidence of postoperative delirium was lower (p = 0.001) in the dexmedetomidine group compared to the ketamine and placebo groups. Delirium occurred in one patient (5%) in the dexmedetomidine group, two patients (10%) in the ketamine group, and 15 patients (75%) in the placebo group (Table 2). After adjusting for all predictors, the dexmedetomidine group had a 32% lower incidence of postoperative delirium than the placebo group (reference) (OR = 0.684, 95% CI: 0.240–0.97, p = 0.025). In contrast, the ketamine group had three times the risk of postoperative delirium as the placebo group (OR = 3.012, 95% CI: 1.185–9.68, p = 0.013). The multivariable logistic regression model of the independent predictors of postoperative delirium revealed that male patients were significant predictors of developing postoperative delirium as they had four times the risk of postoperative delirium as females (OR = 3.950, 95% CI: 1.461–6.064, p = 0.003) (Table 3). However, surgery duration, anesthetic duration, extubation time, and age were not found to be statistically significant predictors of POD.

**Table 2:** Primary and secondary outcomes

	Placebo group (n = 20)	Dexmedetomidine group (n = 20)	Ketamine group (n = 20)	P-value
Incidence of delirium	15 (75%)	1 (5%)	2 (10%)	< 0.001
Incidence of cognitive dysfunction	7 (35%)	0 (0%)	2 (10%)	= 0.006
Time to 1st postoperative request for analgesia (hours)				
	2.30 ± 0.9	2.50 ± 0.9	4.10 ± 1.1	< 0.001
P-value	I vs. II = 0.522	II vs. III < 0.001	I vs. III < 0.001	
The total 24-hour nalbuphine consumption (mg)				
	9.25 ± 1.7	8.40 ± 1.6	6.30 ± 1.4	= 0.006
P-value	I vs. II = 0.356	II vs. III = 0.025	I vs. III = 0.002	
Length of hospital stay (days)				
	4.55 ± 0.6	5.95 ± 0.5	6.15 ± 0.3	= 0.032
P-value	I vs. II = 0.034	II vs. III = 0.757	I vs. III = 0.016	

**Table 3:** Multivariable logistic regression analysis of the independent predictors of postoperative delirium

	OR (95% CI) *	P-value
Age/years	0.984 (0.827–1.171)	= 0.875
Gender (Male)	3.950 (1.461–6.064)	= 0.003
Surgery Duration/min.	0.974 (0.948–1.001)	= 0.055
Anesthesia Duration/min.	1.050 (0.830–1.328)	= 0.687
Extubation Time/min.	0.980 (0.832–1.154)	= 0.809
Treatment Group		
Placebo	1 (Reference)	= 0.046
Dexmedetomidine	0.684 (0.240–0.971)	= 0.025
Ketamine	3.012 (1.185–9.681)	= 0.013

OR = Odds Ratio; CI, Confidence Interval

The incidence of postoperative cognitive dysfunction was lower (p = 0.006) in the dexmedetomidine group compared to the ketamine and placebo groups. No patients reported cognitive dysfunction in the dexmedetomidine group compared to two patients (10%) in the ketamine group and seven patients (35%) in the placebo group (Table 2). After adjusting for all predictors, the effect of the study drugs on the incidence of postoperative cognitive dysfunction was 62% lower in the dexmedetomidine group compared to the placebo group (reference) (OR = 0.375, 95% CI: 0.091–0.543, p = 0.012). In contrast, the ketamine group had 4.5 times the risk of postoperative cognitive dysfunction (OR = 4.501, 95% CI: 1.161–8.817, p = 0.006) when compared to the placebo group (reference). The multivariable logistic regression model of the independent predictors of postoperative cognitive dysfunction revealed that surgery duration, anesthetic duration, extubation time, age, and gender were not found to be statistically significant predictors of postoperative cognitive impairment (Table 4).

**Table 4:** Multivariable logistic regression analysis of the independent predictors of postoperative cognitive dysfunction

	OR (95% CI) *	P-value
Age/years	0.948 (0.828–1.084)	= 0.434
Gender (Male)	0.397 (0.126–1.246)	= 0.113
Surgery Duration/min.	1.074 (0.816–1.914)	= 0.125
Anaesthesia Duration/min.	1.112 (0.771–1.592)	= 0.457
Extubation Time/min.	1.180 (0.655–1.337)	= 0.519
Treatment Group		
Placebo	1 (Reference)	= 0.001
Dexmedetomidine	0.375 (0.091–0.543)	= 0.012
Ketamine	4.501 (1.161–8.817)	= 0.006

*OR = Odds Ratio; CI, Confidence Interval

**Fig. 2:**
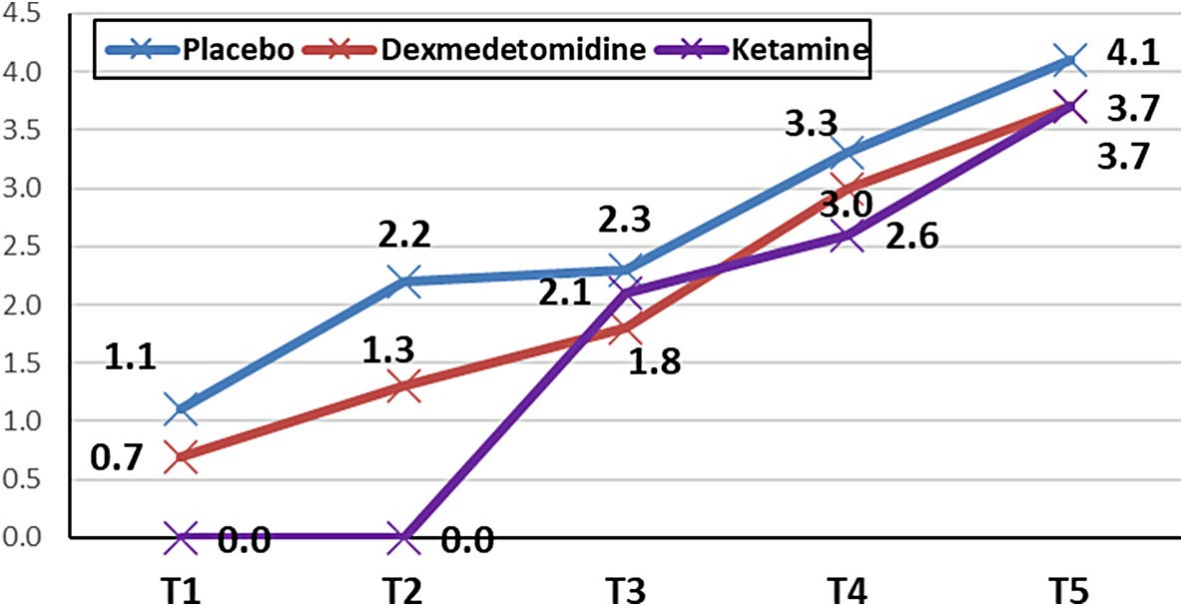
VAS score differences among the studied groups over time

Postoperative pain scores were lower in the ketamine group (0.00 ± 0.0, 0.00 ± 0.0) at 30 and 60 min after surgery compared to the placebo (1.1 ± 0.2, p < 0.001; 2.3 ± 0.1, p < 0.001) and dexmedetomidine (0.7 ± 0.2, p = 0.001; 1.8 ± 0.2, p < 0.001) groups. However, the dexmedetomidine group had lower VAS scores (1.30 ± 0.2; 1.75 ± 0.1) than the placebo group (2.20 ± 0.1, p < 0.001; 2.30 ± 0.2, p = 0.022) at 60 min and 4 h after surgery (Fig. 2). Additionally, the ketamine group had a longer time to first postoperative request for analgesia and a lower postoperative nalbuphine requirement than the placebo (p < 0.001, p = 0.002) and dexmedetomidine (p < 0.001, p = 0.025) groups, respectively.

The length of hospital stay was shorter in the placebo group than in the dexmedetomidine (p = 0.034) and ketamine (p = 0.016) groups. In contrast, there was no significant difference in the length of hospital stay between the ketamine and dexmedetomidine groups (p = 0.757) (Table 2).

## Discussion

In the context of preventing postoperative delirium and cognitive dysfunction in elderly patients, we found that patients who received dexmedetomidine had the lowest incidence of postoperative delirium and cognitive dysfunction compared to ketamine and placebo. A single pre-induction dosage of dexmedetomidine reduced the incidence of postoperative delirium by 32% and cognitive dysfunction by 62% compared to the placebo. However, ketamine use increased the risk of postoperative delirium three times and cognitive dysfunction 4.5 times more than placebo.

Notably, abnormally high levels of pro-inflammatory markers, abnormal neurotransmitter levels, physiologic stresses, and metabolic derangements are the most important pathophysiologic contributors to postoperative delirium [11, 12]. A recent meta-analysis found that proper postoperative pain control is the most effective strategy for reducing POD occurrence in elderly patients undergoing surgery [13]. Dexmedetomidine is a novel sedative-analgesic with neuroprotective properties. Numerous trials have indicated that dexmedetomidine suppresses neuroendocrine and systemic inflammatory processes in response to surgery and significantly lowers proinflammatory cytokines interleukin-6 (IL-6), IL-8, and tumor necrosis factor-alpha (TNF-), as well as microglial activation and neuroapoptosis [14, 15]. Likewise, growing experimental and clinical evidence suggests that ketamine may be neuroprotective because it inhibits NMDA-receptor activation and excitotoxic signaling, as well as decreasing neuronal apoptosis (programmed cell death) [16]. Several studies have demonstrated that intraoperative ketamine has an anti-inflammatory impact, significantly lowering circulating concentrations of the proinflammatory cytokine IL-6 and raising concentrations of the anti-inflammatory cytokine IL-10 following surgery [17, 18]. Ketamine additionally helps to minimize postoperative pain and opioid consumption [19]. Our clinical trial aimed to determine whether a pre-anesthetic bolus of ketamine or dexmedetomidine could reduce the incidence of postoperative delirium and cognitive dysfunction in elderly patients undergoing emergency surgery.

The results from this study are generally in line with a previous meta-analysis of 108 RCTs on delirium pharmacotherapy, which confirmed the effectiveness of dexmedetomidine in reducing the incidence of delirium in postoperative patients (OR 0.46, 95% CI 0.32–0.66, high strength of evidence) [20]. Furthermore, a recent meta-analysis of 21 randomized controlled trials (RCTs) concluded that dexmedetomidine decreased the occurrence of delirium (risk ratio [RR] = 0.55; 95% CI = 0.45 to 0.67) in elderly surgical patients [21]. More studies concluded that perioperative dexmedetomidine administration significantly reduced the incidence of POD in elderly patients undergoing non-cardiac surgery compared to placebo [22–24]. Deiner et al. [25], on the other hand, found that dexmedetomidine did not affect delirium incidence compared to placebo (12.2% [23 of 189] vs. 11.4% [23 of 201], P = 0.94) in elderly patients after noncardiac surgery. This discrepancy could be attributed to differences in treatment drug dosage and timing, as well as differing anesthetic regimens. Furthermore, Lee et al. [26] found that dexmedetomidine dose and timing appeared to be crucial in delirium in elderly patients after laparoscopic major non-cardiac surgery. They discovered that a preoperative dexmedetomidine bolus followed by an infusion reduced the incidence and duration of delirium when compared to a dexmedetomidine bolus administered at the end of surgery. Earlier studies investigated the potential efficacy of a perioperative low dose (0.1 µg/kg/hour) dexmedetomidine infusion on the incidence of POD. Su et al. [27] found that using a low dose dexmedetomidine infusion for elderly patients admitted to intensive care units after non-cardiac surgery reduced the occurrence of postoperative delirium in the dexmedetomidine group (32 [9%] of 350 patients) compared to the placebo group (79 [23%] of 350 patients; odds ratio [OR] 0·35, 95% CI 0·22–0·54; p < 0·0001). In contrast, another study revealed no significant difference in delirium incidence between the dexmedetomidine group and the control group with a low dose dexmedetomidine infusion during anesthesia until the second postoperative day in elective living-donor liver transplant (9% vs. 5.9%; P = 0.44) [28]. Discrepancies in results may be related to variances in the severity of the preoperative disease between patients in the two studies, as well as the different surgical procedures.

Our study showed that ketamine raised the risk of postoperative delirium three times more than placebo. In line with our findings, Hollinger et al. [29] discovered no proof that ketamine helped avoid postoperative delirium or cognitive impairment in the Baden PRIDe study. In addition, a retrospective analysis of 138 patients undergoing spine correction surgery revealed that intraoperative ketamine administration increased the incidence of postoperative delirium compared to the no-ketamine group (OR: 9.475, 95% CI: 1.026–87.508, P = 0.047) [30]. Six randomized controlled studies demonstrated that the incidence of POD did not differ significantly between intraoperative ketamine administration and no intervention in adults undergoing surgery under general anesthesia [31]. Avidan et al. [32] found no difference in postoperative delirium incidence between those who received intraoperatively low dose ketamine (0.5 mg/kg), high dose ketamine (1 mg/kg), or placebo (19.45% vs. 19.82%, respectively; absolute difference, 0.36%; 95% CI, − 6.07–7.38%; p = 0.92). In contrast to the previous unfavorable outcomes with ketamine, Hudetz et al. [18] found in an earlier trial that an intravenous bolus of ketamine (0.5 mg/kg) during anesthetic induction reduced postoperative delirium by 30% following heart surgery. Furthermore, postoperative C-reactive protein concentrations in ketamine-treated patients were significantly lower (p < 0.05) than in placebo-treated patients, indicating that ketamine’s anti-inflammatory activity may be responsible for the neuroprotective impact in this trial.

The results of our study showed that patients in the ketamine group had a long time to first postoperative request for analgesia and less postoperative nalbuphine requirement than the placebo and dexmedetomidine, which could be attributed to ketamine’s analgesic impact [19]. Importantly, the 2016 guidelines on postoperative pain prevention advocate considering intraoperative ketamine as an analgesic adjuvant [33]. Patients who received ketamine had a shorter length of surgery, anesthesia duration, and time to extubation compared to dexmedetomidine and placebo. However, these variables were not statistically significant predictors of POD or POCD. Our data found that the length of hospital stay was shorter in the placebo group than in the dexmedetomidine and ketamine groups. This outcome could be attributable to the fact that patients underwent different surgical procedures with significantly varying postoperative care.

Our research has some limitations. First, it is a single-center study with a small sample size limited to elderly patients scheduled for emergency surgery; therefore, it cannot be extended to all surgical patients. Second, the intraoperative hemodynamic data were collected but not analyzed because the study groups were not sufficiently powered to detect statistically significant changes in hemodynamic parameters. Third, the delirium assessment could only be done twice a day on a regular basis. This could contribute to an underestimation of the incidence of delirium; also, details on the duration and severity of delirium, as well as the treatment offered to delirium patients, were not gathered. Lastly, patients in the study underwent surgeries of several disciplines with greatly different postoperative treatments.

In summary, a considerable body of evidence supports the use of a single pre-induction bolus of dexmedetomidine as a practical choice for preventing postoperative delirium and cognitive dysfunction in elderly patients undergoing emergency surgery.

## Data Availability

All data used and analyzed in this study are available from the corresponding authors upon reasonable request.

## Abbreviations

POD: postoperative delirium
POCD: postoperative cognitive dysfunction
DOSS: Delirium Observation Screening Scale
MMSE: Mini-Mental State Examination
VAS: Visual Analog Scale
ASA: American Society of Anesthesiologists
BMI: Body Mass Index
